# Mediators of negative content and voice‐related distress in a diverse sample of clinical and non‐clinical voice‐hearers

**DOI:** 10.1111/bjc.12396

**Published:** 2022-10-07

**Authors:** Jessica Helen Silver, Marcus Lewton, Heledd Wyn Lewis

**Affiliations:** ^1^ Older Persons Mental Health Psychology Service Cwm Taf Morgannwg University Health Board Pontypridd UK; ^2^ Child and Adolescent Mental Health Service Cwm Taf Morgannwg University Health Board Pontypridd UK; ^3^ South Wales Doctoral Programme in Clinical Psychology Cardiff University Cardiff UK

**Keywords:** auditory verbal hallucinations, distress, hearing voices, mindfulness, thought suppression

## Abstract

**Objectives:**

Negative content in hearing voices (i.e., auditory verbal hallucinations) has been associated with adverse clinical outcomes including voice‐related distress. Voice appraisals and responding mindfully to voices are theorized to reduce voice‐related distress. This study aimed in examine mediators of the negative content voice‐related distress relationship in clinical (those who recently received input from mental health services) and non‐clinical voice‐hearers.

**Methods:**

One hundred and twenty‐one adults (71.9% female; 35.5% mixed or non‐white ethnic background) who hear voices were recruited online and completed self‐report measures of negative content of voices, voice‐related distress, mindfulness of voices, interpretation of loss of control, thought suppression and intrusion.

**Results:**

Clinical voice‐hearers had significantly higher levels of negative content, voice‐related distress and interpretation of loss of control than non‐clinical voice‐hearers. A mindful approach to voices and interpretation of loss of control mediated the relationship between negative content and voice‐related distress across the whole sample. Thought suppression and intrusion did not mediate the relationship.

**Conclusions:**

The results support the use of mindfulness‐based psychological intervention to reduce voice‐related distress. Further development of valid and reliable measures specifically relating to constructs of voice content, voice‐related distress and voice suppression will support further research in this area.

**Practitioner points**

- Negative voice content and voice‐related distress are significantly higher among voice‐hearers who have been in contact with mental health services in the last 6 months.
- Mindfulness of voices and the interpretation of loss of control mediates the relationship between negative voice content and voice‐related distress. This relationship is demonstrated in an ethnically diverse sample.
- Although conclusions about causation cannot be drawn from the findings, they do support the theoretical underpinnings of mindfulness‐based approaches to reducing voice‐related distress.
- These findings indicate that there are several factors that contribute to the maintenance of voice‐related distress. Consideration of the need for a variety of psychological interventions for voice‐related distress is discussed.

## INTRODUCTION

Hearing voices, or auditory verbal hallucinations (AVHs), are conceptualized to exist on a continuum of normal human experience (DeRosse & Karlsgodt, 2015). A high proportion of voice‐hearers do not have a psychiatric diagnosis (Baumeister et al., 2017). It is estimated that up to 10% of the population will hear voices at some point in their lives (British Psychological Society, 2017). The prevalence of hearing voices in the general population has led to the hypothesis that it is not the presence of a voice that is distressing but other factors that lead to distress (Mawson et al., 2010). However, AVHs are considered a core symptom of psychiatric diagnoses that are conceptualized as residing along the schizophrenia spectrum (American Psychiatric Association, 2013; Arciniegas, 2015). These are characterized as severe and enduring, and are associated with high suicide rates, poverty and loss of functioning (Bentall & Morrison, 2002). Understanding factors that protect against psychological distress associated with hearing voices is important to inform clinical interventions (Brett et al., 2014; Johns et al., 2014).

Research has demonstrated that the negative content of voices (e.g., derogatory remarks) is an important factor in clinical outcome. Negative content is associated with voice‐related distress and higher rates of depression (Smith et al., 2006), as well as increased need for care in voice‐hearers (Beavan & Read, 2010; Johns et al., 2014). Voice content is a key difference between clinical and non‐clinical voice‐hearers with clinical voice‐hearers (i.e., those in need of treatment) reporting predominantly negative voice content (Baumeister et al., 2017; Johns et al., 2014; Larøi et al., 2012). In an experience sampling study, those requiring inpatient treatment rated their voices as more negative than those who had not sought treatment (Ben‐Zeev et al., 2020). Furthermore, Rosen et al. (2018) found negative voice content mediates the relationship between childhood adversity and voice‐related distress.

The impact of negative content has also been demonstrated in a simulation study. In an experimental study using a simulation paradigm of AVHs, negative voices increased subjective levels of stress in healthy individuals immediately after exposure, and significantly more than when compared to neutral or ambient noise (Baumeister et al., 2019). Furthermore, in the negative voice condition, more mindful appraisals of the voices were associated with lower levels of subjective stress. Although this study provides important experimental information regarding the role of negative voice content in contributing to distress, there are qualities to the experience of AVHs that are difficult to simulate, such as coming from an unknown origin and being personal in nature.

Despite the above findings, the importance of negative content has been largely neglected in the cognitive model of voices which focuses on beliefs and appraisals of voices (Larøi et al., 2019). Arguably, negative content is closely linked to beliefs about voices as subjective and contextual interpretations are needed for AVH content to be experienced as negative (Larøi et al., 2019).

The cognitive model of voice‐hearing proposes that emotional and behavioural responses are influenced by an individual's appraisal of the voices (Chadwick & Birchwood, 1994; van der Gaag et al., 2003). A systematic review found appraisals of malevolence, supremacy, personal nature and attitudes of rejection and disproval towards the voice‐hearer were most commonly associated with higher levels of distress (Mawson et al., 2010). The review highlighted the limited research on attitudes of approval and acceptance of voices, their influence on distress and the potential links between these attitudes and the hearer's behavioural response. Further research has demonstrated that the appraisal of malevolence is associated with the behavioural response of resistance, and the appraisal of benevolence is associated with engagement with voices (Peters et al., 2012). Overall, in the Peters et al. (2012) study, appraisals were the biggest determinant of behavioural response and distress, independent of the severity of voices. A time‐series network approach study found the presence of voices predicted uncontrollable thoughts in a sample of 95 participants with severe voice‐hearing (Jongeneel et al., 2020).

The Interpretation of Voices Inventory was developed to expand the measurement of beliefs and appraisals relating to voice‐hearing, encapsulating interpretations of loss of control, metaphysical beliefs and positive beliefs about voices (Morrison et al., 2002). Interpretations of voices have been significantly associated with voice characteristics (such as volume and duration), as well as voice‐related distress (Morrison et al., 2004). Specifically, this study found that an increased appraisal of loss of control from voices and endorsement of metaphysical beliefs (e.g., believing voices are a sign of being evil) were associated with higher levels of distress in voice‐hearers. Subsequent research has supported this finding in a larger sample of 101 voice‐hearers (Varese et al., 2016).

Several studies have looked at more general cognitive processes within a psychological flexibility model (Hayes et al., 2011), which is thought to influence the maintenance of distress associated with hearing voices. Experiential avoidance, the negative appraisal of internal experiences leading to attempts to escape or suppress, has been examined for its association with voice‐related distress (Morris et al., 2014; Varese et al., 2016). Varese et al. (2016) found high levels of experiential avoidance, as well as metaphysical beliefs about voices, predicted increased voice‐related distress independent of voice frequency and duration. In contrast, an earlier study demonstrated experiential avoidance was associated with depression and anxiety symptoms but not voice‐related distress specifically (Morris et al., 2014). This pattern of association was also found for non‐judgemental acceptance, an element of mindful behaviour. Furthermore, voice‐related distress in the Morris et al. (2014) study was associated with voice appraisal of malevolence, supporting the cognitive model of voice‐related distress. However, as Varese et al. (2016) point out, these divergent findings may be a product of methodological differences and a small sample size (*N* = 50) in the Morris et al. (2014) study being unable to detect small effects.

Mindful awareness of psychotic symptoms, including hearing voices, has been suggested as a way of developing a different relational approach to symptoms by lessening often habitual unhelpful responses such as engagement or resistance (Abba et al., 2008). General mindfulness capability has been explored in voice‐hearers and is negatively correlated with voice‐related distress (Úbeda‐Gómez et al., 2015) and dysfunctional relationships with the voices (Perona‐Garcelán et al., 2017). When specifically looking at mindfully relating to voices, as measured by the Southampton Mindfulness of Voices Questionnaire, higher levels have been associated with lower voice‐related distress (Dudley et al., 2018) and subjective distress at the time of hearing voices (Chadwick et al., 2007). Increased mindfulness of voices was also associated with lower levels of voice‐related distress and less dysfunctional behavioural responses in a sample of voice‐hearers with psychiatric diagnoses (Stephanie et al., 2018).

Although the above studies show an association of increased mindfulness and lower levels of distress, the directionality cannot be inferred from the cross‐sectional designs. Lower levels of distress could, for example, facilitate more mindful awareness in voice‐hearers. Dudley et al. (2018) found mindfulness of voices mediated the relationship between self‐compassion and severity of voices, but, to a more significant degree, self‐compassion mediated the relationship between mindfulness of voices and severity of voices. It is important to note this study examined mediating influences on severity of voices, rather than voice‐related distress. Experts by experience have highlighted that reducing the negative impact on wellbeing whilst hearing voices, rather than elimination of voice‐hearing all together, is a more important clinical outcome (Corstens et al., 2014).

Theoretical frameworks propose that mindfulness may be helpful for voice‐hearers through several processes, one being that mindful acceptance replaces suppression or experiential avoidance which maintains voice‐related distress (Strauss et al., 2015). Separately, Larøi et al. (2019) propose a theoretical model where negative voice content is reinforced by attempts to suppress, which results in increased voice‐related distress. Both thought suppression and the experience of perceiving thoughts as intrusions, as measured by the White Bear Suppression Inventory, have been associated with susceptibility to hearing voices in large non‐clinical samples (Alderson‐Day et al., 2019; McCarthy‐Jones & Fernyhough, 2006). However, the exploration of thought suppression in populations of people who hear voices and its potential role in maintaining distress has been limited.

Considering the evidence that negative voice content is associated with voice‐related distress, but little is known about the process that may underpin this relationship, there is a need to examine potential mediating factors that may be maintaining distress. Identifying strong mediators in this relationship could elicit therapeutic targets for reducing distress associated with hearing voices. Furthermore, variation may exist in these mediators between clinical and non‐clinical voice‐hearers.

**FIGURE 1:**
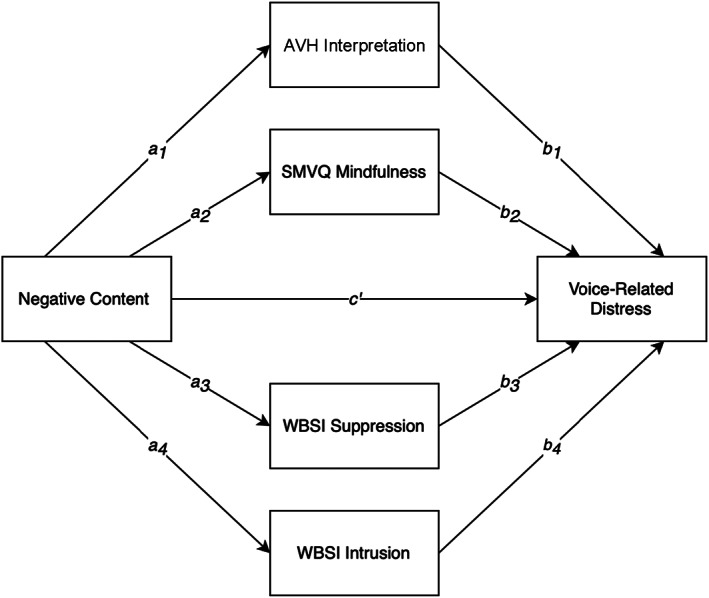
Proposed parallel mediation model showing direct effect pathways

This research aims to examine negative voice content and voice‐related distress in a community sample of voice‐hearers both with and without the need for care. It employs a cross‐sectional design to examine potential mediating facotrs of the voice content and distress relationship, specifically: the role of mindfulness of voices, though supression, intrusions and the interpretation of loss of contol. This study examines the following hypotheses:

1. Clinical voice‐hearers will report a significantly higher level of negative voice content and voice‐related distress than non‐clinical voice‐hearers.
2. Clinical voice‐hearers will report significantly higher interpretations of loss of control, suppression and intrusion but lower levels of mindfulness of voices than non‐clinical voice‐hearers.
3. Level of negative voice content will be positively correlated with level of distress in the combined clinical and non‐clinical sample.
4. Mindfulness, interpretation of loss of control, suppression and intrusion will all mediate the relationship between negative content and voice‐related distress in the combined clinical and non‐clinical sample (see Figure 1).

## METHOD

### Participants

One hundred and seventy‐five adults who hear voices consented to take part in the study by completing an online consent form. Fifty‐four were excluded as they completed less than 90% of the study questions and/or had spent less than 5 min completing the study (an estimated minimum time needed to read and answer all the questions). Completion time is a reliable indicator of meaningless data on internet‐based questionnaires (Leiner, 2019). Data from 121 participants were included for further analysis.

For part of the analysis, participants were divided into two groups, *clinical* (*n* = 75) and *non‐clinical* (*n* = 46). Allocation to the clinical group was determined based on participants having received support from mental health services in the last 6 months. Data were gathered on self‐identified mental health condition or diagnosis. However, recent contact with mental health services was deemed a more suitable indicator of current mental health functioning as a diagnosis could be historical. Demographic and clinical characteristics for the total sample and clinical and non‐clinical group are shown in Table 1.

**TABLE 1:** Demographic and clinical characteristics of participants

	Total sample (*N* = 121)	Clinical group (*n* = 75)	Non‐clinical group (*n* = 46)
*Age*			
18–25	66 (54.5%)	36 (48%)	30 (65.2%)
26–35	32 (26.4%)	21 (28%)	11 (23.9%)
36–45	11 (9.1%)	9 (12%)	2 (4.3%)
46–55	7 (5.8%)	6 (8%)	1 (2.2%)
56–65	5 (4.1%)	3 (4%)	2 (4.3%)
*Sex*			
Female	87 (71.9%)	55 (73.3%)	32 (69.6%)
Male	28 (23.1%)	18 (24%)	10 (21.7%)
Not stated	6 (5%)	2 (2.7%)	4 (8.7%)
*Ethnicity*			
White British	48 (39.7%)	35 (46.7%)	13 (28.3%)
White Irish	2 (1.7%)	2 (2.7%)	0 (0%)
White other	28 (23.1%)	23 (30.7%)	5 (10.9%)
Mixed White and Black Caribbean	1 (.8%)	0 (0%)	1 (2.2%)
Mixed White and Black African	1 (.8%)	1 (1.3%)	0 (0%)
Mixed White and Asian	5 (4.1%)	2 (2.7%)	3 (6.5%)
Mixed other	11 (9.1%)	3 (4%)	8 (17.4%)
Indian	1 (.8%)	0 (0%)	1 (2.2%)
Pakistani	0 (0%)	0 (0%)	0 (0%)
Bangladeshi	0 (0%)	0 (0%)	0 (0%)
Chinese	7 (5.8%)	1 (1.3%)	6 (13%)
Asian other	4 (3.3%)	1 (1.3%)	3 (6.5%)
Black African	5 (4.1%)	2 (2.7%)	3 (6.5%)
Black Caribbean	2 (1.7%)	2 (2.7%)	0 (0%)
Black other	0 (0%)	0 (0%)	0 (0%)
Arab	1 (.8%)	0 (0%)	1 (2.2%)
Other ethnic group	5 (4.1%)	3 (4%)	2 (4.3%)
*Employment status*			
Student	43 (35.5%)	24 (32%)	19 (41.3%)
Unemployed	26 (21.5%)	19 (25.3%)	7 (15.2%)
Retired	3 (2.5%)	2 (2.7%)	1 (2.2%)
Employed	49 (40.5%)	30 (40%)	19 (41.3%)
*Socio‐economic classification*			
Managerial/administrative/professional	27 (22.3%)	15 (20%)	12 (26.1%)
Intermediate occupations	8 (6.6%)	4 (5.3%)	4 (8.7%)
Small employers and own account workers	2 (1.7%)	2 (2.7%)	0 (0%)
Lower supervisory and technical	2 (1.7%)	1 (1.3%)	1 (2.2%)
Semi‐routine and routine occupations	11 (9.1%)	8 (10.7%)	3 (6.5%)
*Mental health condition/diagnosis*			
No	33 (27.3%)	5 (6.7%)	28 (60.9%)
Yes	88 (72.7%)	70 (93.3%)	18 (39.1%)
Schizophrenia	31 (25.6%)		
Anxiety	29 (24%)		
Depression	27 (22.3%)		
PTSD	17 (14%)		
Personality disorder	14 (11.6%)		
Bipolar disorder	11 (9.1%)		
Eating disorder	10 (8.3%)		
Psychosis	8 (6.6%)		
OCD	7 (5.8%)		
Autism	5 (4.1%)		
ADHD	4 (3.3%)		
Substance abuse/addiction	3 (2.5%)		
Phobia	1 (.8%)		

### Measures

A study‐specific demographic information questionnaire was used to obtain age, gender, ethnicity, employment status and information relating to clinical group assignment.

*The National Statistics Socio‐economic Classification* (NS‐SEC; Rose & Pevalin, 2003) self‐coded version was used to determine socio‐economic classification of working or previously employed participants. It differentiates between five classes (see Table 1).

*The Auditory Vocal Hallucination Rating Scale Questionnaire* (AVHRS‐Q; Steenhuis et al., 2019) is a 17‐item self‐report measure that gives an overall severity score for AVHs (ranging from 0, mild to 14, severe). The measure contains fifteen 4‐ and 5‐point Likert scale items and two 10‐point scale items. The measure covers several characteristics including frequency, duration, location, volume, negative content, anxiety and interference with daily functioning. The AVHRS‐Q has been translated into several languages as well as the English version used in this study. The Dutch version has demonstrated good internal consistency (α = .78–.87) and is strongly correlated (*r* = .90) with the AVHRS (a structured interview which the AVHRS‐Q is based on) and measures AVH severity distinct from general psychological distress (Steenhuis et al., 2019). In the current study, the AVHRS‐Q demonstrated good internal consistency (α = .80).

A score of negative content was produced by combining items 9 and 10 from the AVHRS‐Q, which rate content on a scale from always positive to always negative and the degree of unpleasantness to negative content. Scores range from 0, always positive, to 8, highly negative. Internal consistency for the negative content measure was acceptable (Spearman–Brown Coefficient = .67). A voice‐related distress score was produced by combining items 11, 16 and 17 on the AVHRS‐Q. These relate to voices causing anxiety or fear, frequency of bothersome voices and severity of suffering. Scores range from 0, no distress, to 22, high level of voice‐related distress. Internal consistency for the voice‐related distress measure was good (α = .79).

The *Interpretation of Voices Inventory* (Morrison et al., 2002) examines the beliefs that voice‐hearers hold about their voices and includes subscales of metaphysical, positive and loss of control. The loss of control subscale (referred to as AVH interpretation) was the only subscale used and comprises of five 4‐point Likert scale items rating the interpretation of loss of control because of hearing voices (e.g., they mean I will lose control of my behaviour). Total score ranges from 5, no interpretation of loss of control, to 20, high endorsement of loss of control. The subscale has demonstrated internal consistency, α = .88 and good test–retest reliability, *r* = .77 (Byrne & Morrison, 2010). In the current study, internal consistency was good (α = .90).

The *White Bear Suppression Inventory* (WBSI; Wegner & Zanakos, 1994) is a 15‐item self‐report measure, originally developed to assess peoples' tendency to suppress thoughts. Subsequent studies have suggested the measure contains at least two constructs (see Schmidt et al., 2009, for a review). As well as suppression, it most consistently captures a construct of intrusions which relates to the difficulty in controlling unwanted thoughts through the perceived experience of intrusive thoughts. This study utilized the factor structure proposed by Schmidt et al. (2009), which takes into account previous findings across studies using the WBSI in several different languages. They propose two subscales; five items relating to suppression and four items relating to intrusion. In this format, the WBSI has a score range on the suppression subscale of 5 to 25 (high endorsement of thought suppression) and on the intrusion subscale of 4 to 20 (high endorsement of intrusions). With this version of the WBSI, the suppression and intrusion subscales both have good internal consistency, α = .78 and .84, respectively (Schmidt et al., 2009). Internal consistency in this study was good for both suppression (α = .87) and intrusion (α = .85).

The *Southampton Mindfulness of Voices Questionnaire* (SMVQ; Chadwick et al., 2007) measures the degree to which individuals respond mindfully to the voices they hear. It has twelve 7‐point Likert scale items. The measure covers four linked elements of mindfulness; mindful observation, letting go, absence of aversion and non‐judgement. The scale has a total score range of 0 to 72, with higher scores representing a higher degree of tendency to respond mindfully to voices. The SMVQ has shown good internal reliability (α = .84) and moderate concurrent validity with general mindfulness measures (Chadwick et al., 2007). In this study, internal consistency was good (α = .86).

### Procedure

Ethics approval was granted by a University Ethics Committee. Recruitment was carried out online by sharing a digital recruitment poster on a study‐specific Twitter account and by third‐party mental health organizations sharing the recruitment poster on their social media platforms. The eligibility criteria (hearing voices in the last 6 months, 18 years and over, fluent in English) were distributed with the link to the full study information, consent form and questionnaires, which were all hosted on the online secure platform Qualtrics. There was no required severity or frequency of voices. No limitations on geographical location of participants were included in the eligibility criteria due to the online platforms used to promote the study having an international audience. Recruitment was conducted from June to November 2020 when a sufficient sample size for the planned analysis had been reached. To achieve .8 power in percentile bootstrap mediation analysis, Fritz and MacKinnon (2007) recommend a sample size of at least 78 when both *a* and *b* path effect sizes are medium (.39), and a sample of 162 when effect sizes are halfway between small and medium (.26).

### Statistical analyses

Analyses were carried out using IBM SPSS Statistics (version 26) for Mac. Due to multiple tests of difference being carried out, the Benjamini–Hochberg Procedure (Benjamini & Hochberg, 1995) was applied resulting in an α level of .005. A Hotelling's *T* ^2^ test was used to examine the difference between the clinical and non‐clinical groups on all measures. This test enables reporting on the difference between two groups on the combined means of two or more measures as well the difference in individual measure means. A parallel mediation analysis (model 4) was carried out using the PROCESS Macro version 3.5 (Hayes, 2017) to explore the mediating factors of negative content and distress. 95% percentile bootstrap confidence intervals, based on 5000 bootstrap samples, were used to test for an indirect relationship via key variables. An indirect relationship is demonstrated when both confidence intervals are entirely above zero.

## RESULTS

Demographic factors of age, sex, ethnicity and socio‐economic classification were examined for their association with severity of voices, negative content and voice‐related distress; there was no significant associations.

### Difference analysis

Mean scores for all cognitive variables and voice‐related measures for the clinical and non‐clinical groups are provided in Table 2. Prior to carrying out Hotelling's *T* ^2^ test of difference, assumptions were assessed.

**TABLE 2:** Scores on all variables for the clinical and non‐clinical groups

Variable	Clinical		Non‐clinical	
	Range	*M* (*SD*)	Range	*M* (*SD*)
AVHRS‐Q	0–14	6.96 (3.80)	0–14	3.67 (3.72)
Negative content	0–8	5.10 (1.91)	0–8	3.60 (2.03)
Voice‐related distress	0–22	12.06 (5.46)	0–22	7.95 (6.15)
AVH interpretation	5–20	10.70 (4.79)	5–20	8.17 (3.47)
SMVQ mindfulness	7–65	32.96 (14.30)	11–65	40.19 (12.19)
WBSI suppression	12–30	25.52 (4.51)	12–30	23.38 (5.46)
WBSI intrusion	8–30	24.46 (5.29)	15–30	23.10 (4.68)

Abbreviations: AVHRS‐Q, The Auditory Vocal Hallucination Rating Scale Questionnaire; AVH Interpretation, The Interpretation of Voices Inventory Loss of Control Subscale; SMVQ, The Southampton Mindfulness of Voices Questionnaire; WBSI, The White Bear Suppression Inventory.

There was a statistically significant difference between the clinical and non‐clinical groups on the combined study variables (*F*[7, 105] = 3.365, *p* = .003; Wilks' Λ = .817; partial η^2^ = .183).

Hypothesis 1 Severity of voices, negative content and voice‐related distress were significantly higher in the clinical group compared with the non‐clinical group (all *p* < .0005).

Hypothesis 2 AVH interpretation scores were significantly higher in the clinical group compared with the non‐clinical group (*p* = .003). There was no statistically significant difference between the groups on measures SMVQ (*p* = .007), WBSI intrusion (*p* = .168) and suppression (*p* = .026).

### Mediation analysis

A parallel mediation analysis examined whether the relationship between negative voice content and distress was mediated by AVH interpretations, SMVQ, WBSI Suppression and WBSI Intrusion, as illustrated in the proposed mediation model (Figure 1). Prior to analysis, assumptions specific to mediation were checked (see Appendix A). Outliers were detected using Mahalanobis distance, Cook's distance and Leverage and were excluded if they met the criteria for two or more (Aiken et al., 1991). Two cases were identified as being outliers meaning 119 datasets were included in the mediation analysis. All predictor variables were correlated (see Table 3).

**FIGURE 2:**
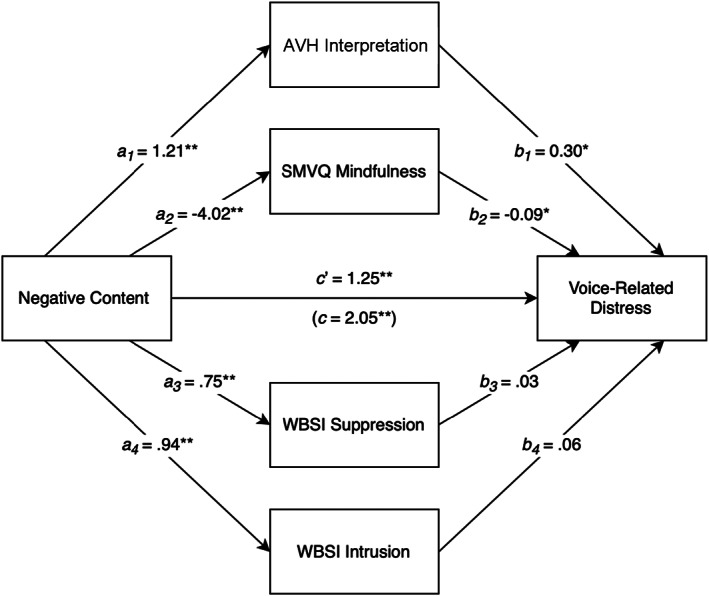
Results of parallel mediation analysis. Figure shows direct effects of negative content on mediator variables (*a*_1‐4_), mediator variables on voice‐related distress (*b*_1‐4_) and negative content on voice‐related distress (*c*′), as well as total effect of negative content on voice‐related distress (*c*); *p < .05; **p < .001.

Hypothesis 3 Parallel mediation analysis found a positive total effect of negative content on voice‐related distress (*c* = 2.05, *p* = <.001). Negative content positively correlated with voice‐related distress. Figure 2 displays total and direct effects.

Hypothesis 4 Parallel mediation analysis found negative voice content indirectly influenced distress in the voice‐hearer through its effect on both interpretation of loss of control (AVH interpretation) and mindfulness (SMVQ). No indirect effect was found for the WBSI subscales of intrusion and suppression. Table 4 displays the indirect effects and confidence intervals for all mediator variables.

**TABLE 3:** Pearson's correlation for all predictor variables (*N* = 111)

Variable	Negative content	AVH interpretation	SMVQ	WBSI suppression
AVH interpretation	.557**			
SMVQ mindfulness	−.598**	−.672**		
WBSI suppression	.333**	.431**	−.397**	
WBSI intrusion	.382**	.537**	−.486**	.723**

Abbreviations: AVH Interpretation, The Interpretation of Voices Inventory Loss of Control Subscale; SMVQ, The Southampton Mindfulness of Voices Questionnaire; WBSI, The White Bear Suppression Inventory.

** *p* < .01 (two tailed).

**TABLE 4:** Indirect effects and confidence intervals

	Indirect effects^a^	95% percentile bootstrap confidence intervals (based on 5000 bootstrap samples)	
AVH interpretation	.36	.10	.64
SMVQ mindfulness	.37	.07	.73
WBSI suppression	.02	−.15	.23
WBSI intrusion	.05	−.21	.31

Abbreviations: AVH Interpretation, The Interpretation of Voices Inventory Loss of Control Subscale; SMVQ, The Southampton Mindfulness of Voices Questionnaire; WBSI, The White Bear Suppression Inventory.

^a^ Unstandardized indirect effects expressed as unit change on Voice‐Related Distress scale.

High levels of negative voice content were associated with a higher degree of interpretation of loss of control (*a*_1_ = 1.21, *p* < .001), and participants with a higher degree of interpretation of loss of control reported increased voice‐related distress (*b*_1_ = .30, *p* = .0147). AVH interpretation exerts an effect of a .36‐point increase on the voice‐related distress scale for every 1‐point increase on the negative content scale.

High levels of negative voice content were associated with less endorsement of mindfulness of voices (*a*_2_ = −4.02, *p* < .001). Lower mindfulness of voices was associated with higher levels of voice‐related distress (*b*_2_ = −.09, *p* = .0195). Mindfulness of voices exerts an effect of a .37‐point increase on the voice‐related distress scale for every 1‐point increase on the negative content scale.

The overall mediation model was statistically significant (*p* < .001) and explained 61% of the variation in voice‐related distress. However, negative content influenced voice‐related distress independent of the indirect effects via the mediator variables examined (*c*′ = 1.25, *p* < .001).

## DISCUSSION

### Summary of findings

The primary aim of this study was to investigate whether there was a difference between clinical and non‐clinical groups on negative content, voice‐related distress, mindfulness, interpretations of control, intrusion and suppression. The secondary aim was to investigate the relationship between negative content and distress in the sample as a whole; specifically, whether mindfulness, interpretations of control, suppression and intrusion mediate the negative content—distress relationship.

This study demonstrated a difference between clinical and non‐clinical groups, with the clinical group reporting higher severity of voices, negative content, voice‐related distress and interpretations of loss of control. This is in line with previous findings that demonstrate differences in negative voice content between clinical and non‐clinical voice‐hearers (Baumeister et al., 2017; Johns et al., 2014; Larøi et al., 2012).

Higher levels of negative content in voices were associated with higher levels of voice‐related distress, supporting previous findings (Rosen et al., 2018; Smith et al., 2006). When this association was explored further, the relationship was mediated by mindfulness and interpretation of loss of control. Mindfulness of voices has previously been associated with lower levels of subjective distress in non‐voice‐hearers exposed to a simulation of negative voices (Baumeister et al., 2019). This study builds on these findings, confirming this pattern in voice‐hearers across a spectrum of severity and level of need. The finding that interpretation of loss of control mediates the relationship between negative content and distress increases the understanding from previous research that it is a predictor of voice‐related distress (Varese et al., 2016).

Thought suppression and intrusion were not mediators. This was unexpected for thought suppression as theoretically it could be considered the opposite of mindfulness, which is characterized as acceptance and non‐judgemental observation of mental activity (Strauss et al., 2015). In a previous study, mindfulness of voices predicted lower levels of resistance to voices (a similar construct to suppression), as well as lower levels of distress (Stephanie et al., 2018). Potentially, suppression and intrusion, and their implications for voice‐related distress, differ between those who recently started hearing voices and those who experienced them for a whilst. However, it is worth noting that the WBSI suppression and intrusion subscales relate specifically to thoughts, not voices. It could be that people who hear voices have differing tendencies for suppressing thoughts in comparison with suppression of mental activity experienced as AVHs. This may be an important differentiating point in future research that continues to build on theoretical models such as that proposed by Larøi et al. (2019) where suppression is implicated in voice‐related distress. Although thought suppression and intrusions have been associated with susceptibility to hearing voices (Alderson‐Day et al., 2019; McCarthy‐Jones & Fernyhough, 2006), exploring these variables in voice‐hearers would benefit from measurement tools that are valid and reliable for this population.

### Clinical implications

Although the cross‐sectional design of this study limits the conclusions that can be drawn about the cause of voice‐related distress, the findings that mindfulness mediates the relationship between negative content and voice‐related distress support the emerging evidence base for the use of mindfulness‐based therapeutic interventions for people with distressing voices (Strauss et al., 2015). A meta‐analysis concluded mindfulness‐based interventions have a small but significant effect in reducing psychosis severity (Louise et al., 2018). The research on mindfulness‐based interventions specifically for distress associated with hearing voices, rather than psychosis, is less developed but shows promising results. A recent randomized controlled trial has shown a group mindfulness‐based intervention to be effective at reducing voice‐related distress, improving perceived controllability of voices and promoting recovery compared with treatment as usual (Chadwick et al., 2016). A small pilot study of an individual mindfulness‐based interventions has demonstrated small to moderate effects in reducing the negative impact of voices (Louise et al., 2019). The findings from these studies demonstrate mindfulness can have a positive effect on distress associated with hearing voices. However, previous research found mindfulness is not associated with level of functioning in voice‐hearers, suggesting mindfulness alone may not be sufficient in improving broader outcomes for voice‐hearers (Morris et al., 2014; Stephanie et al., 2018).

The present study also suggests that interventions based on the cognitive model of voice‐related distress, such as cognitive behavioural therapy for psychosis (CBTp), may be helpful in reducing voice‐related distress if beliefs and appraisals such as the interpretation of loss of control are targeted. However, although CBTp is effective in reducing risky behaviour associated with compliance with command hallucinations by targeting beliefs about the power of voices (Trower et al., 2004), there is limited evidence that CBTp alone is effective in reducing voice‐related distress (Birchwood et al., 2014).

Third‐wave behavioural interventions build upon the well‐established cognitive behavioural model by integrating approaches of acceptance, mindfulness and compassion. Third‐wave approaches such as acceptance and commitment therapy (ACT) may offer a promising alternative to CBT for voice‐related distress. However, their specific utility in reducing distress in psychosis and hearing voices is yet to be clearly demonstrated. A meta‐analysis found ACT to have negligible and non‐significant effects on reducing psychosis symptoms (Louise et al., 2018). Furthermore, although an acceptance‐based CBT approach for command hallucinations has positive outcomes, there is no increased benefit from this intervention on outcomes when compared to an active control of befriending (Shawyer et al., 2012).

Overall, this study supports offering a range of therapeutic interventions to voice‐hearers. Further research on interventions for hearing voices would benefit from including a comparison between psychological approaches, rather than just comparing to treatment as usual.

### Strengths and limitations

Limitations exist around the use of select items from the AVHRS‐Q to measure negative content and voice‐related distress, which have not previously been examined for validity and reliability for this purpose. To our knowledge, there is currently no validated self‐report measure of negative content in voices, and as pointed out by Larøi et al. (2019), it is a difficult construct to measure objectively. Single and two‐item measures for distress and negative content from the Psychotic Symptoms Rating Scales (PSYRATS), an interviewer‐administered measure with items on a 5‐point Likert scale, have been widely used in previous research (Morris et al., 2014; Rosen et al., 2018; So et al., 2016; Stephanie et al., 2018; Varese et al., 2016). The AVHRS‐Q differs in that it is a self‐report measure which enables ease of use in online research. Furthermore, the measures for distress and negative content in this study were made up of several items giving a greater score range (0 to 22 for distress, 0 to 8 for negative content) than using items from the PSYRATS. Nonetheless, a further exploration of the construct validity and development of more robust measures would be beneficial for further research examining voice‐related distress.

The measures were chosen to target key areas hypothesized to be influential in the development of voice‐related distress. A limitation was there was no validity question included to detect inattention in participants. Measures were kept to a minimum to reduce the time taken for participation. However, there are potentially confounding variables that this study did not account for, such as current functioning, coping and medication use. In the mediation analysis, it is important to note that in this study mindfulness and interpretation of loss of control, only partially mediated the effect voice content has on distress. Indicating there are other potentially clinically relevant variables that mediate the relationship between content and distress. Furthermore, possible confounding variables, such as mood, were not controlled for within the mediation analysis. Participants were recruited during the first year of the COVID‐19 pandemic where restrictions, illness and bereavement relating to the pandemic may have influenced levels of distress. However, as information on these factors was not collected this cannot be determined from the data.

The cross‐sectional design of this study means conclusions about directionality cannot be inferred. For example, increased levels of distress in voice‐hearers could facilitate less mindful responding to the voices. Further research with longitudinal data is needed to extrapolate the direction of this relationship. Experience sampling methods have been demonstrated to provide a valid form of longitudinal data collection in voice‐hearers (Ben‐Zeev et al., 2020).

The allocation to clinical and non‐clinical groups was based on a question that was designed to reflect the need for care over the last 6 months. However, it could be that because of the care the clinical group participants received, at the time of undertaking the study, they were coping well. Conversely, there may have been participants in the non‐clinical group that needed care but had not received it for various reasons (e.g., service inaccessibility). Various approaches have been taken to distinguish between clinical and non‐clinical voice‐hearers; however, these often do not account for fluctuations in mental health. Furthermore, the division into groups creates a false dichotomy at odds with the conceptualization of a continuum of mental health.

A strength of this study was the moderate‐to‐large‐sized and ethnically diverse sample. Research being predominantly undertaken with white, young participants limits the applicability to diverse groups. In this study, 35.5% of participants identified as being from a mixed or non‐white ethnic background. Although the sample is biased towards people with internet access, online recruitment is likely to have increased the accessibility of this study to non‐white ethnic groups. However, the sample was skewed towards female, younger adults, which limits the generalizability to older, male voice‐hearers.

## CONCLUSION

Although this study demonstrated differences in voice‐related and AVH interpretation variables between clinical and non‐clinical voice‐hearers, group allocation may not accurately reflect participants' level of functioning. Mediation analysis revealed negative content influences distress indirectly through mindfulness and interpretation of loss of control. These results are demonstrated in an ethnically diverse sample supporting the use of mindfulness‐based interventions for voice‐related distress with diverse populations in clinical practice.

## AUTHOR CONTRIBUTIONS

**Jessica Helen Silver:** Conceptualization; data curation; formal analysis; investigation; methodology; project administration; resources; writing – original draft; writing – review and editing. **Marcus Lewton:** Formal analysis; methodology; supervision; validation; writing – review and editing. **Heledd Wyn Lewis:** Conceptualization; methodology; project administration; supervision; validation; writing – review and editing.

## CONFLICT OF INTEREST

All authors declare no conflict of interest.

## Data Availability

The data that support the findings of this study are openly available in PsychArchives at https://doi.org/10.23668/psycharchives.5654, reference number 5654.

## APPENDIX A

The following assumptions were checked before mediation analysis was carried out: Additivity was checked by calculating bivariate correlations for all predictor variables; all correlation coefficients were less than .80. The assumption of approximate normality was satisfied, as assessed by visual inspection of a histogram. Linearity was checked by visual inspection of a P–P plot and deemed acceptable. The assumption of homoscedasticity was deemed satisfied from visual inspection of a scatterplot of residuals versus predicted values.

Table A1 in this appendix shows bivariate correlation for all variables.

**TABLE A1:** Pearson's correlation for all variables

Variable	AVHRS‐Q	Negative content	Voice‐related distress	AVH interpretation	SMVQ	WBSI suppression
Negative content	.759**					
Voice‐related distress	.844**	.665**				
AVH interpretation	.666**	.557**	.647**			
SMVQ mindfulness	−.669**	−.598**	−.643**	−.672**		
WBSI suppression	.360**	.333**	.358**	.431**	−.397**	
WBSI intrusion	.438**	.382**	.441**	.537**	−.486**	.723**

Abbreviations: AVHRS‐Q, The Auditory Vocal Hallucination Rating Scale Questionnaire; AVH Interpretation, The Interpretation of Voices Inventory Loss of Control Subscale; SMVQ, The Southampton Mindfulness of Voices Questionnaire; WBSI, The White Bear Suppression Inventory.

** *p* < .01 (two tailed).

## References

[bjc12396-bib-0001] Abba, N. , Chadwick, P. , & Stevenson, C. (2008). Responding mindfully to distressing psychosis: A grounded theory analysis. Psychotherapy Research, 18(1), 77–87. 10.1080/10503300701367992 18815958

[bjc12396-bib-0002] Aiken, L. S. , West, S. G. , & Reno, R. R. (1991). Multiple regression: Testing and interpreting interactions. SAGE Publications.

[bjc12396-bib-0003] Alderson‐Day, B. , Smailes, D. , Moffatt, J. , Mitrenga, K. , Moseley, P. , & Fernyhough, C. (2019). Intentional inhibition but not source memory is related to hallucination‐proneness and intrusive thoughts in a university sample. Cortex, 113, 267–278. 10.1016/j.cortex.2018.12.020 30716609 PMC6459394

[bjc12396-bib-0004] American Psychiatric Association . (2013). Diagnostic and statistical manual of mental disorders (5th ed.). American Psychiatric Publishing.

[bjc12396-bib-0005] Arciniegas, D. B. (2015). Psychosis. Continuum (Minneapolis, Minn.), 21, 715–736. 10.1212/01.CON.0000466662.89908.e7 26039850 PMC4455840

[bjc12396-bib-0006] Baumeister, D. , Peters, E. , Pruessner, J. , Howes, O. , & Chadwick, P. (2019). The effects of voice content on stress reactivity: A simulation paradigm of auditory verbal hallucinations. Schizophrenia Research, 243, 225–231. 10.1016/j.schres.2019.07.019 31377050 PMC9205337

[bjc12396-bib-0007] Baumeister, D. , Sedgwick, O. , Howes, O. , & Peters, E. (2017). Auditory verbal hallucinations and continuum models of psychosis: A systematic review of the healthy voice‐hearer literature. Clinical Psychology Review, 51, 125–141. 10.1016/j.cpr.2016.10.010 27866082 PMC5240854

[bjc12396-bib-0008] Beavan, V. , & Read, J. (2010). Hearing voices and listening to what they say: The importance of voice content in understanding and working with distressing voices. Journal of Nervous & Mental Disease, 198(3), 201–205. 10.1097/NMD.0b013e3181d14612 20215997

[bjc12396-bib-0009] Benjamini, Y. , & Hochberg, Y. (1995). Controlling the false discovery rate: A practical and powerful approach to multiple testing. Journal of the Royal Statistical Society: Series B (Methodological), 57(1), 289–300. 10.1111/j.2517-6161.1995.tb02031.x

[bjc12396-bib-0010] Bentall, R. P. , & Morrison, A. P. (2002). More harm than good: The case against using antipsychotic drugs to prevent severe mental illness. Journal of Mental Health, 11(4), 351–356. 10.1080/09638230020023723

[bjc12396-bib-0011] Ben‐Zeev, D. , Buck, B. , Chander, A. , Brian, R. , Wang, W. , Atkins, D. , Brenner, C. J. , Cohen, T. , Campbell, A. , & Munson, J. (2020). Mobile RDoC: Using smartphones to understand the relationship between auditory verbal hallucinations and need for care. Schizophrenia Bulletin Open, 1(1), sgaa060. 10.1093/schizbullopen/sgaa060 33937774 PMC8061119

[bjc12396-bib-0012] Birchwood, M. , Michail, M. , Meaden, A. , Tarrier, N. , Lewis, S. , Wykes, T. , Davies, L. , Dunn, G. , & Peters, E. (2014). Cognitive behaviour therapy to prevent harmful compliance with command hallucinations (COMMAND): A randomised controlled trial. The Lancet Psychiatry, 1(1), 23–33. 10.1016/s2215-0366(14)70247-0 26360400

[bjc12396-bib-0013] Brett, C. , Heriot‐Maitland, C. , McGuire, P. , & Peters, E. (2014). Predictors of distress associated with psychotic‐like anomalous experiences in clinical and non‐clinical populations. British Journal of Clinical Psychology, 53(2), 213–227. 10.1111/bjc.12036 24261699

[bjc12396-bib-0014] British Psychological Society . (2017). In A. Cook (Ed.), Understanding Psychosis and Schizophrenia (revised). British Psychological Society.

[bjc12396-bib-0015] Byrne, R. , & Morrison, A. P. (2010). Young people at risk of psychosis: A user‐led exploration of interpersonal relationships and communication of psychological difficulties. Early Intervention in Psychiatry, 4(2), 162–168. 10.1111/j.1751-7893.2010.00171.x 20536972

[bjc12396-bib-0016] Chadwick, P. , Barnbrook, E. , & Newman‐Taylor, K. (2007). Responding mindfully to distressing voices: Links with meaning, affect and relationship with voice. Tidsskrift for Norsk Psykologforening, 44(5), 581–587.

[bjc12396-bib-0017] Chadwick, P. , & Birchwood, M. (1994). The omnipotence of voices: A cognitive approach to auditory hallucinations. British Journal of Psychiatry, 164(2), 190–201. 10.1192/bjp.164.2.190 8173822

[bjc12396-bib-0018] Chadwick, P. , Strauss, C. , Jones, A. M. , Kingdon, D. , Ellett, L. , Dannahy, L. , & Hayward, M. (2016). Group mindfulness‐based intervention for distressing voices: A pragmatic randomised controlled trial. Schizophrenia Research, 175(1–3), 168–173. 10.1016/j.schres.2016.04.001 27146475 PMC4967452

[bjc12396-bib-0019] Corstens, D. , Longden, E. , McCarthy‐Jones, S. , Waddingham, R. , & Thomas, N. (2014). Emerging perspectives from the hearing voices movement: implications for research and practice. Schizophrenia Bulletin, 40(Suppl_4), S285–S294. 10.1093/schbul/sbu007 24936088 PMC4141309

[bjc12396-bib-0020] DeRosse, P. , & Karlsgodt, K. H. (2015). Examining the psychosis continuum. Current Behavioral Neuroscience Reports, 2(2), 80–89. 10.1007/s40473-015-0040-7 26052479 PMC4454466

[bjc12396-bib-0021] Dudley, J. , Eames, C. , Mulligan, J. , & Fisher, N. (2018). Mindfulness of voices, self‐compassion, and secure attachment in relation to the experience of hearing voices. British Journal of Clinical Psychology, 57(1), 1–17. 10.1111/bjc.12153 28801978 PMC5811822

[bjc12396-bib-0022] Fritz, M. S. , & MacKinnon, D. P. (2007). Required sample size to detect the mediated effect. Psychological Science, 18(3), 233–239. 10.1111/j.1467-9280.2007.01882.x 17444920 PMC2843527

[bjc12396-bib-0023] Hayes, A. F. (2017). Introduction to mediation, moderation, and conditional process analysis, second edition: A regression‐based approach. Guilford Publications.

[bjc12396-bib-0024] Hayes, S. C. , Strosahl, K. D. , & Wilson, K. G. (2011). Acceptance and commitment therapy, second edition: The process and practice of mindful change. Guilford Publications.

[bjc12396-bib-0025] Johns, L. C. , Kompus, K. , Connell, M. , Humpston, C. , Lincoln, T. M. , Longden, E. , Preti, A. , Alderson‐Day, B. , Badcock, J. C. , Cella, M. , Fernyhough, C. , McCarthy‐Jones, S. , Peters, E. , Raballo, A. , Scott, J. , Siddi, S. , Sommer, I. E. , & Larøi, F. (2014). Auditory verbal hallucinations in persons with and without a need for care. Schizophrenia Bulletin, 40(Suppl. 4), S255–S264. 10.1093/schbul/sbu005 24936085 PMC4141313

[bjc12396-bib-0026] Jongeneel, A. , Aalbers, G. , Bell, I. , Fried, E. I. , Delespaul, P. , Riper, H. , van der Gaag, M. , & van den Berg, D. (2020). A time‐series network approach to auditory verbal hallucinations: Examining dynamic interactions using experience sampling methodology. Schizophrenia Research, 215, 148–156. 10.1016/j.schres.2019.10.055 31780345

[bjc12396-bib-0027] Larøi, F. , Sommer, I. E. , Blom, J. D. , Fernyhough, C. , Ffytche, D. H. , Hugdahl, K. , Johns, L. C. , McCarthy‐Jones, S. , Preti, A. , Raballo, A. , Slotema, C. W. , Stephane, M. , & Waters, F. (2012). The characteristic features of auditory verbal hallucinations in clinical and nonclinical groups: State‐of‐the‐art overview and future directions. Schizophrenia Bulletin, 38(4), 724–733. 10.1093/schbul/sbs061 22499783 PMC3406519

[bjc12396-bib-0028] Larøi, F. , Thomas, N. , Aleman, A. , Fernyhough, C. , Wilkinson, S. , Deamer, F. , & McCarthy‐Jones, S. (2019). The ice in voices: Understanding negative content in auditory‐verbal hallucinations. Clinical Psychology Review, 67, 1–10. 10.1016/j.cpr.2018.11.001 30553563

[bjc12396-bib-0029] Leiner, D. J. (2019). Too fast, too straight, too weird: Non‐reactive indicators for meaningless data in internet surveys. Survey Research Methods, 13(3), 229–248. 10.18148/srm/2019.v13i3.7403

[bjc12396-bib-0030] Louise, S. , Fitzpatrick, M. , Strauss, C. , Rossell, S. L. , & Thomas, N. (2018). Mindfulness‐ and acceptance‐based interventions for psychosis: Our current understanding and a meta‐analysis. Schizophrenia Research, 192, 57–63. 10.1016/j.schres.2017.05.023 28545945

[bjc12396-bib-0031] Louise, S. , Rossell, S. L. , & Thomas, N. (2019). The acceptability, feasibility and potential outcomes of an individual mindfulness‐based intervention for hearing voices. Behavioural and Cognitive Psychotherapy, 47(2), 200–216. 10.1017/s1352465818000425 29983128

[bjc12396-bib-0032] Mawson, A. , Cohen, K. , & Berry, K. (2010). Reviewing evidence for the cognitive model of auditory hallucinations: The relationship between cognitive voice appraisals and distress during psychosis. Clinical Psychology Review, 30(2), 248–258. 10.1016/j.cpr.2009.11.006 20071062

[bjc12396-bib-0033] McCarthy‐Jones, S. , & Fernyhough, C. (2006). The role of thought suppression and metacognitive beliefs in proneness to auditory verbal hallucinations in a non‐clinical sample. Personality and Individual Differences, 41, 1421–1432. 10.1016/j.paid.2006.06.003

[bjc12396-bib-0034] Morris, E. M. , Garety, P. , & Peters, E. (2014). Psychological flexibility and nonjudgemental acceptance in voice hearers: Relationships with omnipotence and distress. Australian & New Zealand Journal of Psychiatry, 48(12), 1150–1162. 10.1177/0004867414535671 24835207

[bjc12396-bib-0035] Morrison, A. P. , Nothard, S. , Bowe, S. E. , & Wells, A. (2004). Interpretations of voices in patients with hallucinations and non‐patient controls: A comparison and predictors of distress in patients. Behaviour Research and Therapy, 42(11), 1315–1323. 10.1016/j.brat.2003.08.009 15381440

[bjc12396-bib-0036] Morrison, A. P. , Wells, A. , & Nothard, S. (2002). Cognitive and emotional predictors of predisposition to hallucinations in non‐patients. British Journal of Clinical Psychology, 41(Pt 3), 259–270. 10.1348/014466502760379127 12396254

[bjc12396-bib-0037] Perona‐Garcelán, S. , Rodríguez‐Testal, J. F. , Senín‐Calderón, C. , Ruiz‐Veguilla, M. , & Hayward, M. (2017). Mindfulness as a mediator between the relational style with voices and negative affect. Mindfulness, 8(2), 454–459. 10.1007/s12671-016-0617-6

[bjc12396-bib-0038] Peters, E. R. , Williams, S. L. , Cooke, M. A. , & Kuipers, E. (2012). It's not what you hear, it's the way you think about it: Appraisals as determinants of affect and behaviour in voice hearers. Psychological Medicine, 42(7), 1507–1514. 10.1017/s0033291711002650 22115329

[bjc12396-bib-0039] Rose, D. , & Pevalin, D. (2003). A researcher's guide to the national statistics socio‐economic classification (vol. 52). 10.1016/s0277-9536(00)00136-2 11005397

[bjc12396-bib-0040] Rosen, C. , McCarthy‐Jones, S. , Jones, N. , Chase, K. A. , & Sharma, R. P. (2018). Negative voice‐content as a full mediator of a relation between childhood adversity and distress ensuing from hearing voices. Schizophrenia Research, 199, 361–366. 10.1016/j.schres.2018.03.030 29580740 PMC6151289

[bjc12396-bib-0041] Schmidt, R. E. , Gay, P. , Courvoisier, D. , Jermann, F. , Ceschi, G. , David, M. , Brinkmann, K. , & Van der Linden, M. (2009). Anatomy of the White Bear Suppression Inventory (WBSI): A review of previous findings and a new approach. Journal of Personality Assessment, 91(4), 323–330. 10.1080/00223890902935738 20017061

[bjc12396-bib-0052] Shawyer, F. , Farhall, J. , Mackinnon, A. , Trauer, T. , Sims, E. , Ratcliff, K. , Larner, C. , Thomas, N. , Castle, D. , Mullen, P. , & Copolov, D. (2012). A randomised controlled trial of acceptance‐based cognitive behavioural therapy for command hallucinations in psychotic disorders. Behaviour Research and Therapy, 50(2), 110–121. 10.1016/j.brat.2011.11.007 22186135

[bjc12396-bib-0042] Smith, B. , Fowler, D. G. , Freeman, D. , Bebbington, P. , Bashforth, H. , Garety, P. , Dunn, G. , & Kuipers, E. (2006). Emotion and psychosis: Links between depression, self‐esteem, negative schematic beliefs and delusions and hallucinations. Schizophrenia Research, 86(1–3), 181–188. 10.1016/j.schres.2006.06.018 16857346

[bjc12396-bib-0043] So, S. H. , Begemann, M. J. , Gong, X. , & Sommer, I. E. (2016). Relationship between neuroticism, childhood trauma and cognitive‐affective responses to auditory verbal hallucinations. Scientific Reports, 6, 34401. 10.1038/srep34401 27698407 PMC5048145

[bjc12396-bib-0044] Steenhuis, L. A. , Pijnenborg, G. H. M. , Visser, E. , van de Willige, G. , van Beilen, M. , Nauta, M. H. , Aleman, A. , & Bartels‐Velthuis, A. A. (2019). The development, validity, and reliability of the auditory vocal hallucination rating scale questionnaire (AVHRS‐Q). Social Psychiatry & Psychiatric Epidemiology, 54(8), 927–935. 10.1007/s00127-019-01692-z 30903236

[bjc12396-bib-0045] Stephanie, L. , Susan, L. R. , Wei Lin, T. , Monique, S. , & Neil, T. (2018). Does mindfulness help people adapt to the experience of hearing voices? Psychiatry Research, 270, 329–334. 10.1016/j.psychres.2018.09.013 30292085

[bjc12396-bib-0046] Strauss, C. , Thomas, N. , & Hayward, M. (2015). Can we respond mindfully to distressing voices? A systematic review of evidence for engagement, acceptability, effectiveness and mechanisms of change for mindfulness‐based interventions for people distressed by hearing voices. Frontiers in Psychology, 6, 1154. 10.3389/fpsyg.2015.01154 26321980 PMC4536375

[bjc12396-bib-0047] Trower, P. , Birchwood, M. , Meaden, A. , Byrne, S. , Nelson, A. , & Ross, K. (2004). Cognitive therapy for command hallucinations: randomised controlled trial. British Journal of Psychiatry, 184, 312–320. 10.1192/bjp.184.4.312 15056575

[bjc12396-bib-0048] Úbeda‐Gómez, J. , León‐Palacios, M. G. , Escudero‐Pérez, S. , Barros‐Albarrán, M. D. , López‐Jiménez, A. M. , & Perona‐Garcelán, S. (2015). Relationship between self‐focused attention, mindfulness and distress in individuals with auditory verbal hallucinations. Cognitive Neuropsychiatry, 20(6), 482–488. 10.1080/13546805.2015.1089225 26413817

[bjc12396-bib-0049] van der Gaag, M. , Hageman, M. C. , & Birchwood, M. (2003). Evidence for a cognitive model of auditory hallucinations. The Journal of Nervous and Mental Disease, 191(8), 542–545. 10.1097/01.nmd.0000082183.95853.ec 12972858

[bjc12396-bib-0050] Varese, F. , Morrison, A. P. , Beck, R. , Heffernan, S. , Law, H. , & Bentall, R. P. (2016). Experiential avoidance and appraisals of voices as predictors of voice‐related distress. British Journal of Clinical Psychology, 55(3), 320–331. 10.1111/bjc.12102 26752336

[bjc12396-bib-0051] Wegner, D. M. , & Zanakos, S. (1994). Chronic thought suppression. Journal of Personality, 62(4), 615–640. 10.1111/j.1467-6494.1994.tb00311.x 7861307

